# Regulation of lysosomal ion homeostasis by channels and transporters

**DOI:** 10.1007/s11427-016-5090-x

**Published:** 2016-07-19

**Authors:** Jian Xiong, Michael X. Zhu

**Affiliations:** Department of Integrative Biology and Pharmacology, McGovern Medical School, Program of Cell and Regulatory Biology, Graduate School of Biomedical Sciences, The University of Texas Health Science Center at Houston, Houston 77030, USA

**Keywords:** lysosomal storage disease (LSD), ion homeostasis, calcium, lysosome acidification

## Abstract

Lysosomes are the major organelles that carry out degradation functions. They integrate and digest materials compartmentalized by endocytosis, phagocytosis or autophagy. In addition to more than 60 hydrolases residing in the lysosomes, there are also ion channels and transporters that mediate the flux or transport of H^+^, Ca^2+^, Na^+^, K^+^, and Cl^−^ across the lysosomal membranes. Defects in ionic exchange can lead to abnormal lysosome morphology, defective vesicle trafficking, impaired autophagy, and diseases such as neurodegeneration and lysosomal storage disorders. The latter are characterized by incomplete lysosomal digestion and accumulation of toxic materials inside enlarged intracellular vacuoles. In addition to degradation, recent studies have revealed the roles of lysosomes in metabolic pathways through kinases such as mechanistic target of rapamycin (mTOR) and transcriptional regulation through calcium signaling molecules such as transcription factor EB (TFEB) and calcineurin. Owing to the development of new approaches including genetically encoded fluorescence probes and whole endolysosomal patch clamp recording techniques, studies on lysosomal ion channels have made remarkable progress in recent years. In this review, we will focus on the current knowledge of lysosome-resident ion channels and transporters, discuss their roles in maintaining lysosomal function, and evaluate how their dysfunction can result in disease.

## INTRODUCTION

Similar to the cytosol, lysosomal lumens also contain a pool of various ions, including H^+^, Na^+^, K^+^, Ca^2+^, Cl^−^, Fe^2+^, and Zn^2+^, which exert unique and indispensable physiological functions. H^+^ is believed to be important to maintain the activity of lysosomal digestive enzymes (Mindell, 2012), which may be roughly classified into three groups, glycosidases, proteases and sulfatases, and most of them require an acidic environment to function optimally (Table 1). Ca^2+^ is important for vesicle trafficking (Wong et al., 2012). Na^+^ is required for the function of some lysosomal transporters, such as SLC38 family transporters (Mackenzie and Erickson, 2004; Wang et al., 2015). K^+^ regulates the lysosomal membrane potential and lysosomal Ca^2+^ homeostasis (Cang et al., 2015; Cao et al., 2015b). Cl^−^ serves as a counterion to regulate lysosomal membrane potential and, to some extent, facilitate the acidification of lysosome lumen (Kasper et al., 2005; Lange et al., 2006; Graves et al., 2008). Fe^2+^ catalyzes the hydrolysis of H_2_O_2_ and produces reactive oxygen species (Dixon et al., 2012). Zn^2+^ is a trace element that serves as an essential coenzyme for about 300 proteins (Tapiero and Tew, 2003).

**Figure 1:**
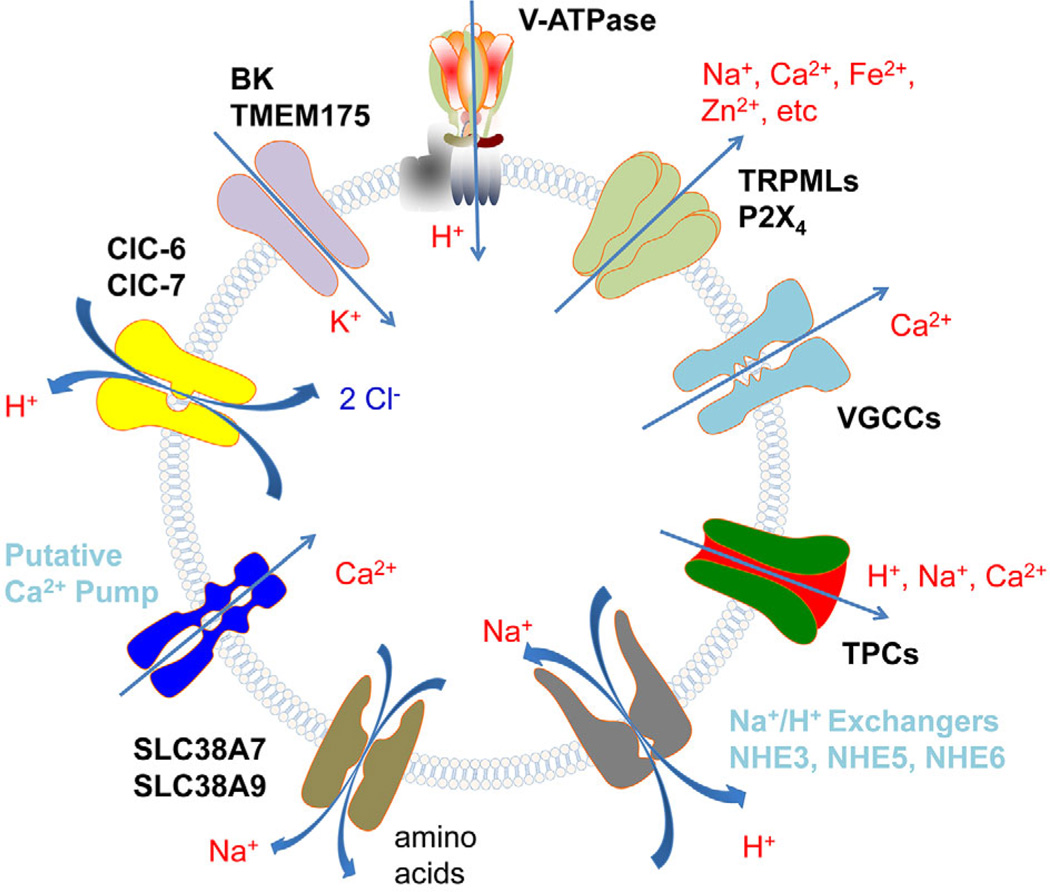
Ion channels and ion transporters on the lysosomal membrane. Both identified and putative players are included. Arrows indicate the direction of ion fluxes. V-ATPase is the proton pump that acidifies lysosome. Confirmed lysosomal channels and transporters include non-selective cation channels (TRPML and P2X_4_) voltage-gated Ca^2+^ channels (VGCC), two-pore channels (TPC) that are permeable to H^+^, Ca^2+^, Na^+^, SLC38 transporters that co-transport Na^+^ and amino acids (SLC38A7 and SLC38A9), ClC transporters that exchange cytosolic Cl^−^ for lysosomal H^+^ (ClC-6 and ClC-7), K^+^ channels (BK and TMEM175). Putative lysosomal ion transporters include Na^+^/H^+^ exchangers (NHE3, NHE5 and NHE6) and Ca^2+^ pump or Ca^2+^/H^+^ exchanger that mediates lysosomal uptake of Ca^2+^ from the cytosol.

The ionic movement across the lysosomal membrane is regulated by a set of ion channels and transporters. To date, for each type of the ions described above, at least one corresponding conductive channel/transporter has been identified and for some, multiple channels/transporters have been shown to be responsible for their transportation across the lysosomal membrane (Figure 1). For example, TRPML proteins form non-selective cation channels that reside on endolysosomal membranes and are permeable to multiple types of positively charged ions, i.e. cations (Dong et al., 2008; Eichelsdoerfer et al., 2010; Feng et al., 2014); Two pore channels, or TPCs (Ishibashi et al., 2000), conduct Na^+^ and Ca^2+^ release from endolysosomes (Calcraft et al., 2009; Wang et al., 2012; Arredouani et al., 2015). ClC-7 serves to transport Cl^−^ across lysosomal membranes (Kasper et al., 2005; Lange et al., 2006; Graves et al., 2008). More recently, the large conductance Ca^2+^-activated K^+^ (BK) channel, previously only known to work on the plasma membrane and mitochondria (Xu et al., 2002; Wang and Tonggu, 2015), was found to reside and function on endolysosomal membranes of several different cell types, especially nonexcitable cells that were not known to possess functional BK channels before (Cao et al., 2015b). Remarkably, BK is unlikely the only K^+^ channel type present on these acidic organelles. A transmembrane protein, TMEM175, has been reported to form a novel lysosomal K^+^ channel (Cang et al., 2015).

For a given ionic type, the amount of ionic flow is determined by the permeability of the channels to that ion and the driving force governed by the electrochemical gradients. It should be reminded that a typical lysosome has ~50-fold higher surface/volume ratio than a regular cell, assuming the diameters of 0.2 and 10 µm for the lysosome and cell, respectively, and both being perfect spheres. This means a more dramatic change in the ionic composition of the lysosomal lumen upon opening or closing of lysosomal channels. The change in ion concentrations will lead to a marked change in lysosomal membrane potential, which can swing, perhaps, even a broader range than that typically found on the plasma membrane of excitable cells. The change in membrane potential in turn will alter the driving force and/or even the permeability, if the affected conductance is voltage sensitive, to other ions. Therefore, it is not surprising that ion channels of the endolysosomal membrane can directly or indirectly affect the activities of each other. Consequently, lysosomal ion channels should be under stringent regulation and ion transporters and pumps should be in place to help maintain the ion homeostasis across the lysosomal membranes.

Furthermore, because ion channels and transporters in the same lysosome share the same membrane potential and ionic pool, functional interplay among channels or transporters situated on the same membrane is common and cellular defects associated with lysosome channel dysfunction also tend to be similar. These include alterations in lysosomal pH (Kasper et al., 2005; Lange et al., 2006; Graves et al., 2008; Lin et al., 2015; Cang et al., 2015), endocytic vesicle trafficking (Wong et al., 2012), autophagy, substance degradation and lysosome exocytosis (Wong et al., 2012; Medina et al., 2015). Therefore, lysosomal ion channels and transporters play critical roles in maintaining proper lysosomal functions essential for cell survival. In the following sections, we will discuss the function and interactions between different lysosomal ion channels and transporters.

## ION HOMEOSTASIS AND ION CHANNELS IN THE LYSOSOMES

### H^+^ (Proton)

Under normal conditions, the pH of lysosomes is maintained at around 4.5, whereas the cytosolic pH is usually neutral. The H^+^ gradient across the lysosomal membrane is generated and maintained by vacuolar H^+^-ATPase, or V-ATPase, which translocates two protons into the lysosome by consuming one ATP (Beyenbach and Wieczorek, 2006). Other transporters, such as Na^+^ and Cl^−^ transporters, utilize the H^+^ gradient to translocate ions in and out of lysosomes (see later). The blockade of V-ATPase by its inhibitor, bafilomycin A1, however, only increases lysosomal pH up to around 6 (Yoshimori et al., 1991), indicating that either residual V-ATPase activity or additional acidification mechanism may exist to keep lysosomes from being completely neutralized.

**Figure 2:**
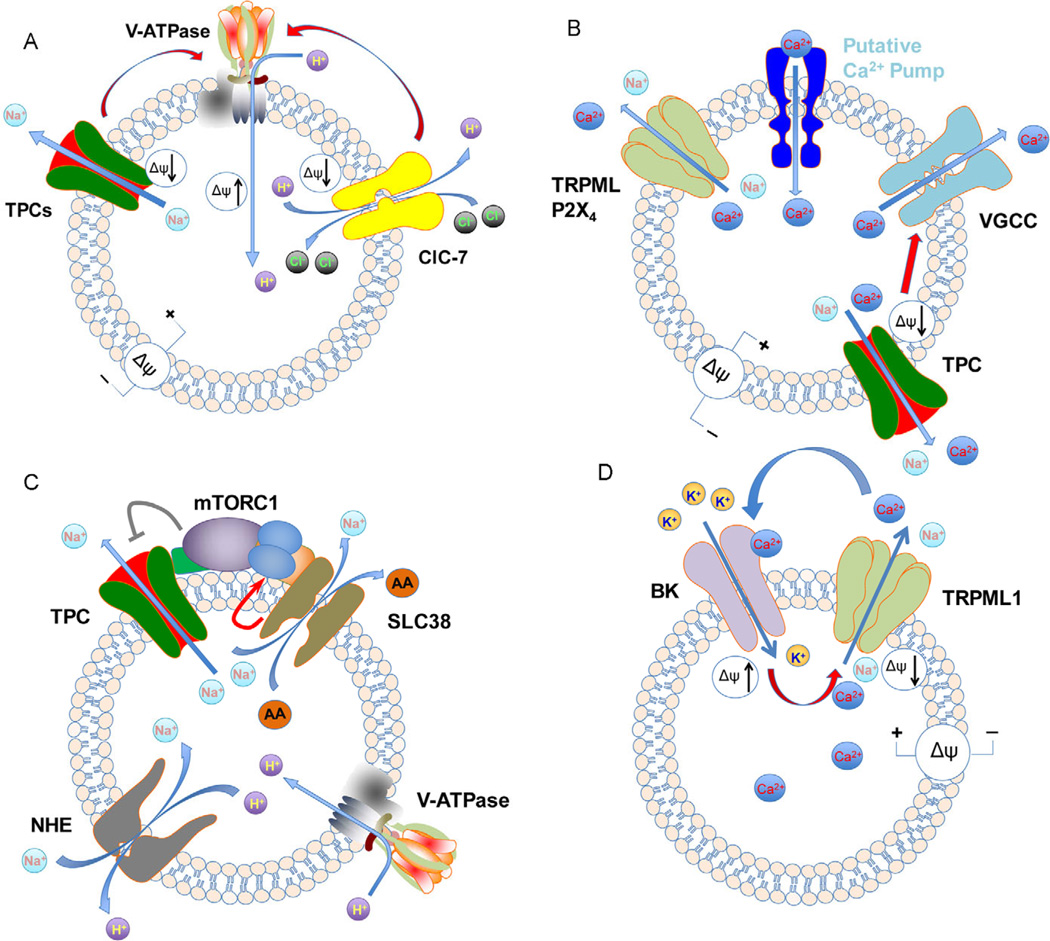
Cooperation of ion channels/transporters in regulation of ion homeostasis. A, Regulation of H^+^ homeostasis by V-ATPase, TPCs and ClC-7. V-ATPase acidifies the lysosomes by pumping H^+^ into lysosome lumen. This also hyperpolarizes the lysosomal membrane. The hyperpolarization is countered by (i) TPCs, which release Na^+^ from the lysosomes and (ii) ClC-7, which exchanges two cytosolic Cl for one luminal H^+^. B, Regulation of Ca^2+^ homeostasis by putative Ca^2+^ transporters, VGCCs, non-selective cation channels and TPCs. A putative Ca^2+^ pump is responsible for lysosomal Ca^2+^ uptake. Nonselective cation channels (TRPMLs, P2X_4_), VGCCs and TPCs can release Ca^2+^ from the lysosome under different conditions. C, Regulation of Na^+^ homeostasis by TPCs, NHEs and SLC38 transporters, and the effect on metabolism. Na^+^ accumulates in the lysosomal lumen most likely due to the combined actions of V-ATPase, NHE, and/or Na^+^/K^+^-ATPase. The luminal Na^+^ fuels the Na^+^-dependent amino acid (AA) transporters, such as SLC38A9, to export AA generated from digestion from lysosomes to the cytosol. AA starvation causes mechanistic target of rapamycin complex 1 (mTORC1) to leave the lysosome. This relieves the blockade of TPCs (Cang et al., 2013), which then dumps Na^+^ out to halt SLC38 transporters, resulting in AA accumulation inside the lysosome. The gradual buildup of AA at the luminal side eventually leads to reactivation of mTORC1 via SLC38A9 (Wang et al., 2015; Rebsamen et al., 2015; Jung et al., 2015), inactivation of TPCs, AA release and finally autophagy termination. D, Positive feedback reinforcement of TRPML1-mediated lysosomal Ca^2+^ release via BK channels. Ca^2+^/Na^+^ efflux through TRPML1 leads to lysosomal membrane depolarization, which lowers the driving force for continued Ca^2+^ release. The activation of BK by depolarization and the cytosolic Ca^2+^ signal generated by TRPML1 causes K^+^ inflow to the lysosome and hyperpolarization, allowing continued Ca^2+^ release through the open TRPML.

It has been shown that disruption of certain channels, for example TPC2, led to abnormal lysosomal pH handling (Lu et al., 2013; Lin et al., 2015) (Figure 2A). However, cautions should be taken with interpretation of results from lysosomal pH measurement because the pH probes used are able to label all acidic organelles, including endosomes, which typically have higher luminal pH than lysosomes (Mindell, 2012). As such, impairment in vesicle trafficking may cause accumulation of the pH probe in endosomes and thereby an increased population of higher pH vesicles. Therefore, some of the experimental results could also be explained by a defect in endolysosomal trafficking. Alternatively, the lower than neutral pH obtained in bafilomycin A1-treated cells could result from residual V-ATPase activity, which only needs to work very little to maintain a somewhat more acidic pH in lysosome lumen than in the cytosol. For instance, a lysosome of 300 nm diameter only needs less than 10 protons to maintain a pH of 6 [1×10^6^ mol L^−1^ H^+^ in a volume of 1.41×10^−17^ L, multiplied by the Avogadro constant, 6.02×10^23^ mol^−1^] as compared to close to 1,000 protons required for pH 4.0.

### Ca^2+^ (Calcium ion)

Lysosomes take up Ca^2+^ from cytosol in a pH dependent manner. In yeast (Cunningham and Fink, 1994; 1996) and plants (Hirschi et al., 1996; Geisler et al., 2000), there are two transporters that move Ca^2+^ into the vacuole, a lysosome equivalent in these organisms. One is a homolog of mammalian plasma membrane Ca^2+^-ATPases (PMCA), which consumes ATP to pump Ca^2+^ into the vacuole. The other is a Ca^2+^/H^+^ exchanger, which transports Ca^2+^ from cytosol to vacuolar lumen in exchange for removal of H^+^. However, although genes related to the yeast/plant Ca^2+^/H^+^ exchanger have recently been described in other vertebrates (Melchionda et al., 2016), no homolog was found in placental mammals, despite the evidence showing pH dependence of lysosomal Ca^2+^ uptake and maintenance in mammalian cells. In mouse macrophages, while neutralizing lysosomes with NH_4_Cl caused a fast release of Ca^2+^ into the cytosol, the removal of NH_4_Cl allowed rapid restoration of high lysosomal Ca^2+^ (Christensen et al., 2002). The H^+^-dependence of lysosomal Ca^2+^ uptake/maintenance indicates a closely coupled homeostatic control of H^+^ and Ca^2+^ contents inside lysosomes also in mammalian cells. However, the molecular basis and functional mechanism(s) of this regulation remain to be elucidated.

Ca^2+^ may also be taken up from extracellular space into the cell via endocytosis. However, a substantial portion of the Ca^2+^ taken up this way is lost from endosomes in exchange for acidification of these organelles (Gerasimenko et al., 1998). Therefore, it is unclear how much of the endocytosed Ca^2+^ actually reaches lysosomes. However, given that lysosomal Ca^2+^ content was reduced by removal of extracellular Ca^2+^ (Christensen et al., 2002), a fraction of the lysosomal Ca^2+^ may come from extracellular space due to fusion with endocytosed cargos.

Different from the limited knowledge on Ca^2+^ uptake into lysosomes, much more is known about Ca^2+^ release from the lysosomes (Figure 2B). One of the best studied lysosomal Ca^2+^ release signals is nicotinic acid adenine dinucleotide phosphate (NAADP). This highly potent Ca^2+^ mobilizing messenger, which typically works at low nanomolar and even high picomolar concentrations, was first discovered in sea urchin eggs (Clapper et al., 1987) and later shown to exist in mammalian cells (Cancela et al., 1999). The NAADP-induced Ca^2+^ release was not affected by depleting the endoplasmic reticulum (ER) Ca^2+^ store with thapsigargin, an inhibitor of the sarco/endoplasmic reticulum Ca^2+^-ATPase (SERCA) (Genazzani and Galione, 1996). However, it is sensitive to treatment with glycyl-L-phenylalanine 2-naphthylamide (GPN) (Churchill et al., 2002), a cathepsin C substrate that disrupts lysosomal membranes by increasing the osmolality and thereby depleting its Ca^2+^ content, as well as bafilomycin A1, the V-ATPase inhibitor described above which indirectly disrupts the lysosomal Ca^2+^ gradient because of the loss of H^+^. These experiments established lysosomes or lysosome-like acidic organelles as the intracellular source of NAADP-induced Ca^2+^ signals.

It was not until more recently that the channels involved in mediating NAADP-induced lysosome Ca^2+^ release were shown to be made of TPCs (Calcraft et al., 2009; Brailoiu et al., 2009; Zong et al., 2009). In human and mouse, there are two isoforms of TPCs, TPC1 and TPC2. Other vertebrates, including most mammals, also have an additional isoform, TPC3. The three TPCs share the same membrane topology with 12 predicted transmembrane (TM) segments clearly segregated into two 6-TM domains, which exhibit sequence similarity to that of voltage-gated Ca^2+^ channels and voltage-gated Na^+^ channels (Calcraft et al., 2009; Rahman et al., 2014). The predicted membrane topology has recently gained experimental support from the crystal structures of *Arabidopsis thaliana* TPC1 (Guo et al., 2016; Kintzer and Stroud, 2016), which is expressed in plant vacuoles and functions as a Ca^2+^-activated Ca^2+^ release channel (Peiter et al., 2005). The *Arabidopsis* TPC1 represents the sole TPC in high plants and is equally distant from mammalian TPC1, TPC2, and TPC3 (Calcraft, et al., 2009).

Using the recently developed whole-endolysosome patch clamp technique, a number of laboratories have demonstrated that TPCs form Na^+^-selective channels that become activated upon stimulation by phosphatidylinositol 3,5-bisphosphate [PI(3,5)P_2_] (Wang et al., 2012; Cang et al., 2013, 2014), an endogenous phosphoinositide mainly found in late endosomes, multivesicular bodies and lysosomes (Li et al., 2013), but not phosphatidylinositol 4,5-bisphosphate [PI(4,5)P_2_], the plasma membrane isotype of the phosphoinositide (Wang et al., 2012; Ruas et al., 2015). In some cases, similar currents were also elicited by NAADP at nanomolar concentrations (Rybalchenko et al., 2012; Grimm et al., 2014; Ruas et al., 2015). At the time of this writing, the Ca^2+^ conductance for the PI(3,5)P_2_-evoked TPC currents under the whole-endolysosome configuration remains uncertain (Wang et al., 2012; Cang et al., 2013; Ruas et al., 2015) and will require further investigation. On the other hand, NAADP-evoked currents have been detected in planar lipid bilayers using crude immunoprecipitants from cells that overexpressed epitope-tagged TPC1 or TPC2. These currents exhibit different electrophysiological properties as that from whole-endolysosome recordings and show conductance not only to Na^+^, but also to H^+^, K^+^ and Ca^2+^ (Pitt et al., 2011, 2014).

Despite the controversy on the biophysical properties and mechanisms of activation of TPCs, recent studies have revealed the importance of these channels in vesicular trafficking associated with endocytosis, autophagy, platelet maturation, and viral infection (Ruas et al., 2010; Pereira et al., 2011; Lu et al., 2013; Lin et al., 2015; Sakurai et al., 2015; Ambrosio et al., 2015). Among them, it is interesting to note that both TPC1 and TPC2 are essential for the entry of Ebola virus from endosomal vesicles to the cytoplasm of the host cells following macropinocytosis. Mouse embryonic fibroblast (MEF) cells derived from TPC1 or TPC2 knockout animals showed resistance to infection of mouse-adapted Ebola virus, suggesting that targeting TPCs may be a good strategy for antiviral therapy (Sakurai et al., 2015).

In excitable cells, plasma membrane depolarization activates voltage-gated Ca^2+^ channels to initiate cytosolic Ca^2+^ concentration elevation, which in turn regulates many physiology processes, including muscle contraction, gene transcription, synaptic transmission and so on (Catterall, 2011, Buraei et al., 2015). A recent study, however, unexpectedly found the presence of the pore-forming α1A subunits of voltage-gated Ca^2+^ channels in lysosomes of both fruit flies and mice (Tian et al., 2015). Mutations of the α1A subunits, as well as that of the auxiliary α2δ2 subunits of the voltage-gated Ca^2+^ channels, disrupted autophagy and the fusion between lysosomes and endosomes and/or autophagic vacuoles in fly photoreceptor cells and mouse cerebellar neurons (Tian et al., 2015). Although it remains to be directly demonstrated the presence of voltage-gated Ca^2+^ currents in lysosomal membranes, the reported study suggests an intriguing possibility that voltage-gated Ca^2+^ channels may be present in lysosomal membranes of many different cell types and function to release Ca^2+^ from the acidic organelles upon lysosomal membrane depolarization. The Ca^2+^ signals generated through this fashion appear to be pivotal for vesicle fusion. If this is true, any channel activity that causes lysosomal membrane depolarization can potentially induce Ca^2+^ release from lysosomes through activation of voltage-gated Ca^2+^ channels. Clearly, the Na^+^-conductive TPCs are top candidates that mediate endolysosome membrane depolarization in response to the rise of PI(3,5)P_2_ and NAADP levels in the cell. However, the functional coupling between TPCs and voltage-gated Ca^2+^ channels has yet to be demonstrated. It is also not known whether the lysosomal voltage-gated Ca^2+^ channels have the same or different subunit compositions and/or splice variants as those typically found on the plasma membranes of excitable cells.

The mucolipin family of proteins represent another group of Ca^2+^-permeable channels that function on the membranes of endolysosomes. The mammalian mucolipin family consists of three non-selective cation channels, TRPML1, TRPML2 and TRPML3, belonging to the large superfamily of transient receptor potential (TRP) channels. Like other members of the TRP channels, each TRPML channel is composed of four subunits, and each subunit contains 6 TM segments (Venkatachalam et al., 2014), resembling, topologically, a single domain of the TPC proteins described above. In both over-expression systems and native cells, TRPML1 is almost exclusively localized to lysosomes, TRPML2 is mostly localized in endosomes (Sun et al., 2015) while TRPML3 is expressed in both lysosomes and plasma membrane (Venkatachalam et al., 2006; Samie et al., 2009; Cheng et al., 2010). In terms of tissue distributions, whereas TRPML1 is widely expressed in most cell types, TRPML2 and TRPML3 expressions are restricted to only certain tissues (Cheng et al., 2010).

TRPML1 was first discovered because its mutations were linked to mucolipidosis type IV (MLIV) (Slaugenhaupt et al., 1999; Bargal et al., 2000; Bassi et al., 2000; Sun et al., 2000), a lysosomal storage disorder (LSD) that impairs neurodevelopment. Multiple loss-of-function mutations in TRPML1 had been identified from MLIV patients and the lysosomal storage phenotype has been recapitulated in animal models in which *TRPML1* gene was deleted (Venkatachalam et al., 2008; Micsenyi et al., 2009). Similar to TPC2, TRPML1 is also activated by PI(3,5)P_2_ (Dong et al., 2010), and its proper function is necessary for a series of endocytic vesicle trafficking events, including lysosome exocytosis (LaPlante et al., 2006), large particle phagocytosis (Samie et al., 2013) and vesicle fusion (Wong et al., 2012). Synthetic TRPML ligands have been identified through high throughput drug screening and lead optimization (Grimm et al., 2010; Shen et al., 2012). Some of these may be of therapeutic potentials as the trafficking deficits and lysosomal storage in cells bearing some MLIV causing mutants, or even other types of LSDs that exhibit impaired TRPML1 function (see below), were rescued by the synthetic TRPML1 agonists (Shen et al., 2012; Chen et al., 2014). TRPML channel activities have been shown to be inhibited by the plasma membrane localized PI(4,5)P_2_, indicating that although TRPML proteins can be found on the plasma membrane, the channels mainly function on endolysosomal membranes (Zhang et al., 2012; Feng et al. 2014).

In cells from another LSD, the Niemann-Pick disease, which results from defective sphingomylinase activity, the accumulated sphingomyelin in the lysosome lumen leads to an inhibition of TRPML1 channel activity. Apparently, the impaired TRPML1 activity may be responsible for the lysosomal storage of this disease as activation of TRPML1 by a synthetic agonist greatly reduced the accumulation of cholesterol in these cells (Shen et al., 2012). Supporting a general role of TRPML1 function in lysosomal storage, another study on Pompe’s disease found that upregulating the expression and activity of TRPML1 through activation of TFEB promoted lysosomal content clearance via lysosome exocytosis (Medina et al., 2011). Conversely, TRPML1-mediated lysosomal Ca^2+^ release was shown to activate the TFEB pathway via calcineurin (Medina et al., 2015), the Ca^2+^-calmodulin regulated phosphatase (Klee et al., 1979). Altogether, these studies not only show an essential role of TRPML1 in maintaining lysosomal health and homeostasis, but also suggest that activating TRPML1 to promote lysosome exocytosis can be a potential strategy to treat multiple types of LSDs.

Compared to TRPML1, much less is known about TRPML2 and TRPML3. Similar to TRPML1, both TRPML2 and TRPML3 can be activated by PI(3,5)P_2_ (Dong et al., 2010). Overexpression of TRPML2 facilitated activation of the small GTPase, Arf6, which regulates recycling of glycosylphosphatidylinositol-anchored proteins (GPI-APs) (Karacsonyi et al., 2007). High expression of TRPML2 was found in the kidney and immune organs and tissues, including thymus, spleen and lymph nodes (Cuajungco et al., 2016). Activating macrophages with Toll-like receptor (TLR) agonists such as lipopolysaccharide (for TLR4) or R848 (for TLR7/8) upregulated TRPML2 mRNA levels. Functionally, TRPML2 knockout mice showed defects in recruiting macrophages to injected bacteria, revealing a role of TRPML2 in the innate immune response (Cuajungco et al., 2016).

A gain-of-function mutation in TRPML3 was identified in varitint-waddler mice, which exhibit deafness and fur pigmentation defects, presumably due to excessively elevated cytosolic Ca^2+^ levels resulting from constitutive channel activation and plasma membrane targeting (Di Palma et al., 2002; Grimm et al., 2007; Kim et al., 2007; Nagata et al., 2008). To some extent, despite the obvious differences in biophysical properties and subcellular localizations (Xu et al., 2007), TRPML1 and TRPML3 may share overlapping or complementing functions. For example, a growth delay in the neonates was observed only in mice with deficiencies in both TRPML1 and TRPML3, but not those with a deficiency in either isoform alone. Detailed histological analysis by electron microscopy revealed that the defect was due to inability of the lysosomes to digest milk proteins, a critical process of nutrient absorption normally carried out by enterocytes in the intestine of suckling pups (Remis et al., 2014). In fact, TRPML3 can form heteromultimeric channels with TRPML1, as the dominant-negative mutant of TRPML1 abolished the conductivity of TRPML3 co-expressed in the same cell (Venkatachalam et al., 2006; Zeevi et al., 2010). On the other hand, because TRPML3 has an opposite pH dependence as compared to TRPML1, being more active at the neutral pH than acidic ones (Kim et al., 2008), the presence of TRPML3-containing channels would allow lysosomes to release Ca^2+^ under conditions when the pH gradient became disrupted because of stress and/or pathogen infection. In the case of bladder epithelial cells, the activation of TRPML3-containing channels in response to lysosome pH neutralization caused by the invading uropathogenic *Escherichia coli* triggers lysosome exocytosis, which expels the exosome-encased bacteria to protect cells from infection (Miao et al., 2015).

**Figure 3:**
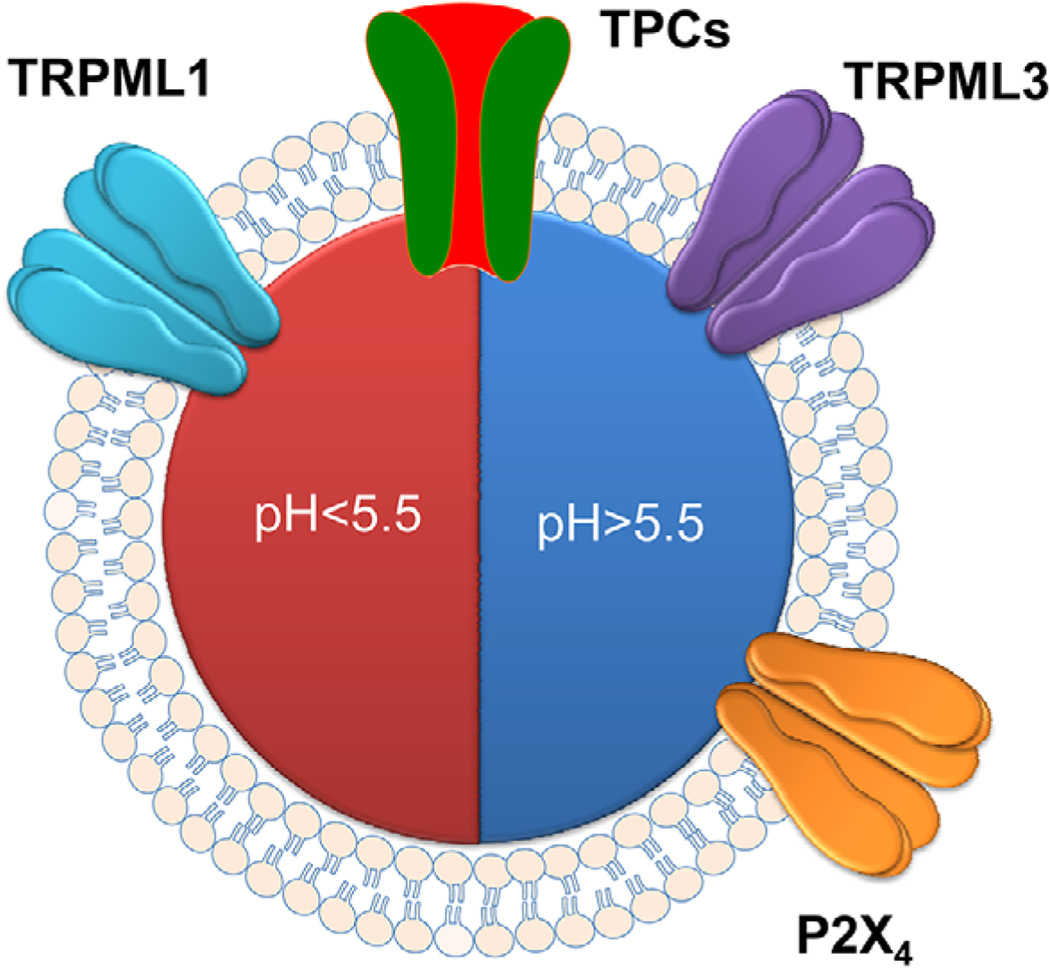
Different pH optima of lysosomal Ca^2+^-permeable channels. For channels that mediate Ca^2+^ release from the lysosomes, TRPML1 works optimally in acidic pH, TRPML3 and P2X_4_ works best at neutral pH, TPCs can work in a broad pH range.

Finally, P2X_4_ is a Ca^2+^-permeable channel widely expressed in many tissues, including the central and peripheral nervous system, epithelial cells, smooth muscle and so on (Bo et al., 2003). Recent studies have shown its localization in lysosomes (Qureshi et al., 2007; Cao et al., 2015a). The lysosomal P2X_4_ channel remains inactive in the acidic lysosomal pH environment, but becomes activated at the neutral pH due to the presence of high ATP content in the lysosomal lumen (Huang et al., 2014). This unique pH-dependent activation of P2X_4_ may explain why neutralizing lysosomes by V-ATPase inhibitors or NH_4_Cl leads to Ca^2+^ release (Christensen et al., 2002) (Figure 3). It was shown that the P2X_4_-mediated lysosome Ca^2+^ release is critical for lysosome fusion in a calmodulin-dependent manner (Cao et al., 2015a).

### Na^+^ (Sodium ion)

Na^+^ probably represents the most abundant cation inside the lysosomes (Wang et al., 2012). How lysosomes take up Na^+^ remains unknown, although three Na^+^/H^+^ exchangers, NHE3, NHE5, and NHE6, have been shown to be expressed in endocytic vesicles (Orlowski and Grinstein, 1997). There are two main functions for Na^+^ in the lysosomes: (i) to regulate lysosomal membrane potential; (ii) to functionally couple to certain amino acid transporters that belong to the SLC38 family of sodium-amino acid co-transporters.

Lysosomal membrane potential, here defined as Δψ=ψ_cytosol_−ψ_lysosome_, is determined by the concentration gradients of all ions between the lysosomal lumen and the cytosol and the relative permeabilities of the lysosomal membrane to these ions. Cation uptake into the lysosome will make the lysosomal membrane potential more negative, i.e. hyperpolarize it, which can counter the pumping action of V-ATPase for lysosome acidification. Therefore, excess Na^+^ in the lumen can hinder lysosomal acidification and as such, the activity of Na^+^-permeable channels can have a significant impact on lysosome pH regulation. Results from studying lysosomal pH of cells from mice lacking the Na^+^-permeable TPC1 and TPC2 channels support this idea. In one case, macrophages isolated from mice deficient in both TPCs exhibited elevated lysosomal pH under starvation conditions (Cang et al., 2013). In another study, primary skeletal muscle cells isolated from TPC2 knockout mice cultured under fed conditions showed a shift of the mean lysosomal acidity by 0.5 pH unit towards neutral (Lin et al., 2015). By contrast, MEF cells isolated from an independent TPC2 knockout mouse line did not display a change in lysosomal pH, even under starved conditions (Grimm et al., 2014). These studies revealed that the Na^+^-permeant TPCs can contribute to lysosomal pH regulation at least under certain conditions or in certain cell types (Figure 2A).

To date, two members of the SLC38 family of sodium and amino acid co-transporters, SLC38A7 and SLC38A9, have been shown to localize on the lysosomal membranes (Hägglund et al., 2011; Chapel et al., 2013; Wang et al., 2015; Rebsamen et al., 2015; Jung et al., 2015). These proteins typically function as Na^+^-dependent amino acid transporters. Of the eleven members in the SLC38 family, four are not well characterized. Therefore, it is possible that more SLC38 transporter subtypes are lysosome-localized. SLC38A1–5 are either amino acid-Na^+^ symporter or amino acid-Na^+^ symporter/H^+^ antiporter (Bröer, 2014). While detailed functional characterization of SLC38A7 and SLC38A9 is largely lacking, the activities of these transporters have been shown to be Na^+^ dependent (Hägglund et al., 2011; Rebsamen et al., 2015). This differs from several other amino acid transporters described on the lysosomal membranes, e.g. cystinosin and SLC36A1 (or PAT1), which are Na^+^-independent (Bröer and Palacín, 2011).

Amino acid transporters not only export the newly generated amino acids from lysosomal digestion to the cytoplasm, but also help regulate amino acid levels inside the lysosomal lumen. The latter function is crucial for the regulation of mTOR complex 1 (mTORC1) (Zoncu et al., 2011). It should be interesting to note a functional interplay among mTORC1, TPCs and SLC38A9 during autophagy, where Na^+^ efflux via TPCs suppresses the Na^+^-dependent transport of amino acids through SLC38A9. This allows accumulation of amino acids, especially arginine, in the lysosomal lumen during early hours of autophagy until they reach the level to trigger mTORC1 reactivation (Wang et al., 2015; Rebsamen et al., 2015; Jung et al., 2015). The activated mTORC1 then inhibits TPCs, presumably by phosphorylation (Cang et al., 2013), allowing the buildup of luminal Na^+^ content presumably from combined actions of V-ATPase and NHEs, or even Na^+^/K^+^-ATPases, which then facilitates amino acid export to support anabolic activities (Figure 2C). Therefore, the dynamic regulation of Na^+^ fluxes across the lysosomal membrane is important for not only lysosomal membrane potential but also Na^+^-dependent solute transport that strongly influences cell metabolism. Supporting this argument, the skeletal muscle from mice deficient in TPC2 exhibited reduced phosphorylated mTOR level and mTORC1-regulated activities, as well as delayed autophagy termination (Lin et al., 2015). In mice that lacked both TPC1 and TPC2, amino acid homeostasis in blood circulation was also disrupted following food deprivation (Cang et al., 2013).

### K^+^ (Potassium ion)

The luminal concentration of K^+^ in endolysosomes is estimated to be lower than that in the cytosol (Wang et al., 2012). By using ultracentrifugation and mass spectrometry, Wang and colleagues had isolated endolysosomes and estimated that the luminal concentration of Na^+^ is ~100-fold higher than that of K^+^ (Wang et al., 2012). This result is different from a previous study using an indirect null-point titration method based on exchange between protons and the monovalent cations with defined stoichiometry in the presence of ionophores, which determined the luminal K^+^ concentration to be ~60 mmol L^−1^ while Na^+^ to be ~20 mmol L^−1^ (Steinberg et al., 2010). However, the study by Wang and colleagues did not rule out the possibility that the basal activity of lysosomal ion channels and transporters could alter the distributions of certain ions during the process of lysosome isolation or the lysosomal preparation might contain fractions of other membrane compartments such as Golgi. On the other hand, the null-point titration assay assumed constant lysosomal membrane permeabilities to Na^+^ and K^+^ that were independent of lysosomal pH changes. Therefore, alternative methods, such as that using selective indicator dyes to measure ion concentrations inside and outside the organelles, are still needed to evaluate Na^+^ and K^+^ concentrations under basal and stimulated conditions. Because of the large pH fluctuations in the lysosomal lumen, these indicators ideally should be relatively pH insensitive.

Thus far, at least two types of K^+^ channels have been identified and functionally characterized in endolysosomal membranes (Cao et al., 2015b; Cang et al., 2015). The K^+^ channels mediate K^+^ influx into the endolysosomal lumen, which in turn hyperpolarizes the endolysosomal membrane. In one case, the presence of the large conductance Ca^2+^-activated K^+^ channels (BK) on endolysosomal membranes and its activation by the very Ca^2+^ released from the organelle via TRPML1 facilitated the continued Ca^2+^ efflux through the open TRPML1 channels (Cao et al., 2015b) (Figure 2D). The influx of K^+^ provides a counter-ion movement to help maintain the membrane potential, which otherwise quickly dissipates because of the loss of Na^+^ and Ca^2+^ and the small volumes of endosomes and lysosomes. It is intriguing that blocking BK recapitulated many of the lysosomal storage phenotypes seen with TRPML1 deficiency and enhancing TRPML1 function abrogated the defects seen due to BK inhibition, but not vice versa (Cao et al., 2015b). This suggests that BK mainly acts through TRPML1 to regulate lysosome functions. In another study, TMEM175, a novel membrane protein with two repeats of the 6-TM segment domains, was shown to form the sole K^+^ conductance of endolysosomal membranes (Cang et al., 2015). Macrophages lacking TMEM175 were shown to have defects in lysosomal acidification and autophagy flux under starvation (Cang et al., 2015). This phenotype is rather similar to that of TPC1 and TPC2 deficient cells (Cang et al., 2013). However, the functional relationship between TPCs and TMEM175 remains to be elucidated.

To date, it has not been made clear how K^+^ is removed from endolysosomes, although the α1 subunit of Na^+^/K^+^-ATPases has been found to be associated with lysosomal membrane proteins in a proteomic study (Lin-Moshier et al., 2014). If a Na^+^/K^+^-ATPase is indeed expressed and functional on the lysosomal membrane, it will exclude K^+^ from and take up Na^+^ into the lysosome. Additionally, nonselective cation channels found on the endolysosomal membranes, such as TRPMLs and P2X_4_, also permeate K^+^. These channels could release K^+^ into the cytosol under conditions when electrochemical gradients favor K^+^ efflux.

### Fe^2+^ (Ferrous ion)

**Figure 4:**
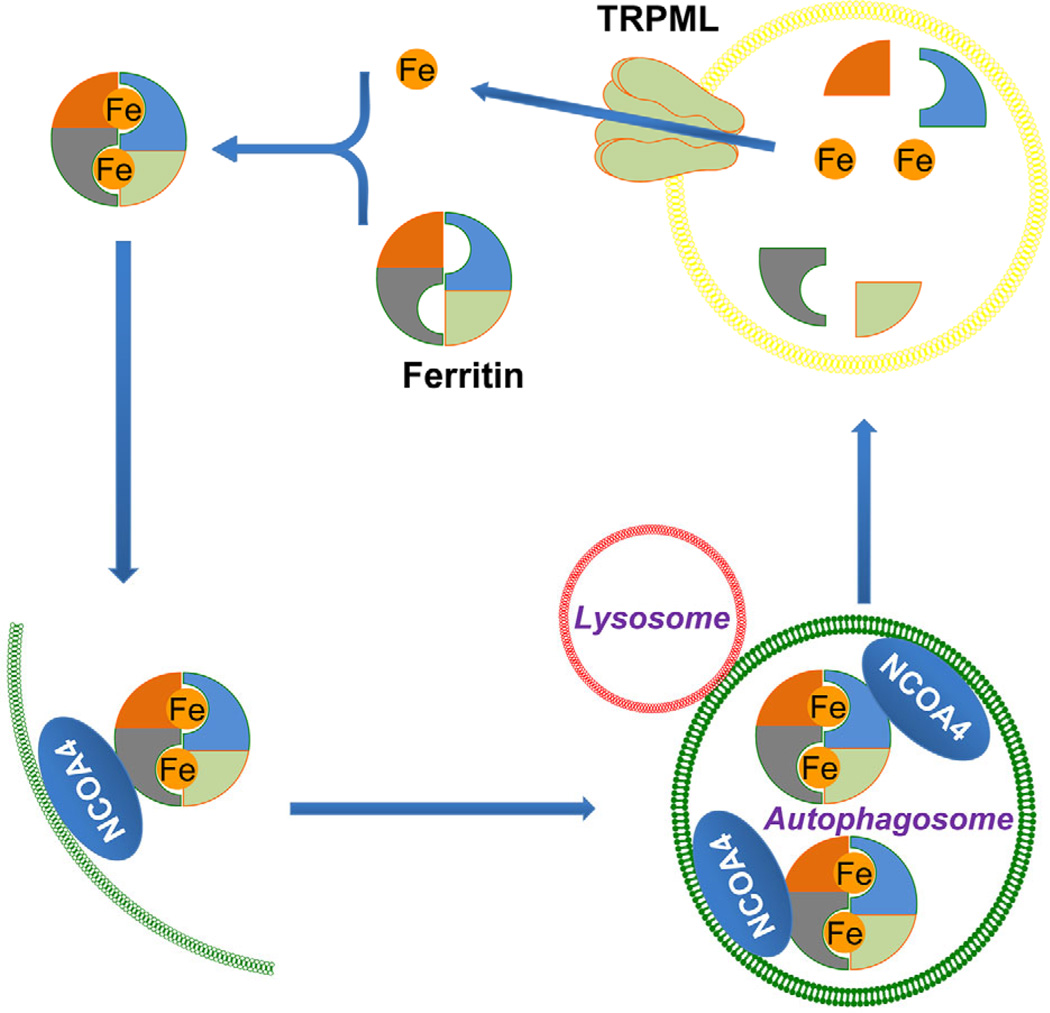
Regulation of cellular Fe^2+^ homeostasis by ferritin and TRPML1. In the cytosol, Fe^2+^ is stored in complex with ferritin. Nuclear Receptor Coactivator 4 (NCOA4) mediates ferritin trafficking to autophagosomes, which then fuse with lysosomes to allow degradation of ferritin and free Fe^2+^ from the bound state. Subsequently, TRPML1 mediates Fe^2+^ efflux from the lysosome to the cytosol.

In the intestine, Fe^3+^ (ferric) taken from food needs to be converted to Fe^2+^ by Dcytb (duodenal cytochrome b reductase) before DMT-1 (divalent cation/metal transporter-1, or DCT-1) on the apical membrane of enterocytes can take it up (Hentze et al., 2004). Fe^2+^ catalyzes the production of reactive oxygen species and therefore is tightly regulated. To keep the Fe^2+^ concentration low in the cytosol, the iron-binding protein, ferritin, is highly expressed in every animal cell type, where 24 subunits of ferritins form a hollow sphere to store Fe^2+^ (Shi et al., 2008). To release the bound Fe^2+^ from ferritins, the nuclear receptor coactivator 4 (NCOA4) is recruited to act as the cargo receptor to deliver ferritin to lysosomes or autophagosomes. After degradation of ferritin, Fe^2+^ is freed (Mancias et al., 2014) and subsequently released from the lysosomal lumen to the cytosol via TRPML1 in mammals and TRPML in flies (Dong et al., 2008; Feng et al., 2014) (Figure 4). In flies, there is only one *trpml* gene (Venkatachalam et al., 2008). In mammals, TRPML2 can also mediate Fe^2+^ release, but the iron permeability of TRPML3 is unclear (Dong et al., 2008).

### Zn^2+^ (Zinc ion)

Disruption of Zn^2+^ homeostasis in the cytosol can potentially lead to growth defects, impaired immune response, diabetes, and neurodegenerative diseases. Therefore, cytosolic Zn^2+^ concentrations need to be tightly regulated. Zn^2+^ is taken up to the lysosome by zinc transporters, ZnT2 and ZnT4 (Palmiter et al., 1996; Huang and Gitschier, 1997; McCormick and Kelleher, 2012). Its release, again, is mediated by TRPML channels. Knocking down TRPML1 in HEK293 cells caused excessive Zn^2+^ storage and dyshomeostasis in the cell (Eichelsdoerfer et al., 2010), revealing a new mechanism of pathology caused by defects in lysosomal ion channels.

### Cl^−^ (Chloride ion)

Cl^−^ is the most abundant anion inside the lysosome (Stauber and Jentsch, 2013). ClC-7 represents the major lysosomal Cl^−^ transporter in all cell types (Graves et al., 2008) while ClC-6 functions as another major lysosomal Cl^−^ transporter in both the central nervous system and peripheral nervous system (Poët et al., 2006). Both transporter isoforms function as Cl^−^/H^+^ exchangers, which move two chloride ions-into the lysosomal lumen in exchange of one H^+^ out (Graves et al., 2008). Because of this uneven exchange, it is thought that the activity of ClC-7 (and ClC-6) helps maintain the lysosomal membrane potential to favor the uptake of H^+^ by V-ATPase (Ishida et al., 2013). Indeed, knocking down ClC-7 in HeLa cells impaired lysosomal acidification (Graves et al., 2008). However, no significant change in lysosomal pH was detected in macrophages, neurons, and embryonic fibroblasts isolated from ClC-7 knockout mice, suggesting that alternative mechanisms also exist to facilitate lysosomal acidification in the absence of this transporter (Kasper et al., 2005; Lange et al., 2006; Steinberg et al., 2010). Nevertheless, gene ablation of either *Clc-6* or *Clc-7* led to lysosomal storage and neurodegeneration (Kasper et al., 2005; Poët et al., 2006); either deleting the *Clc-7* gene or specifically uncoupling its H^+^ and Cl^−^ conductance impaired bone resorption and caused osteoporosis (Kornak et al., 2001; Weinert et al., 2010). Therefore, the critical importance of ClC-7 type of Cl^−^/H^+^ antiporters in lysosome function is well established.

## LYSOSOMAL ION CHANNELS IN CELL METABOLISM

The cellular responses to nutrient deficiency have been well studied. These include a combination of cytosolic and transcriptional events that help the cell adapt to the stressed conditions. Lysosomal ion channels and transporters described above have all been shown to play roles in this process one way or the other. First, nutrient deprivation leads to increased TRPML1 activity, which releases Ca^2+^ from lysosomes (Medina et al., 2015). The Ca^2+^ signal then activates calcineurin, the phosphatase that dephosphorylates TFEB, the master transcriptional factor for lysosome biogenesis. The activation of TFEB allows transcription of a set of genes involved in autophagy and lysosome biogenesis, including subunits of V-ATPase (Sardiello et al., 2009; Settembre et al., 2011) and thereby facilitates lysosomal degradation of nonessential cellular components to support survival.

Second, as a component of mTORC1, TPCs are inhibited by mTOR. Upon nutrient deprivation, this inhibition is relieved, allowing TPC1 and TPC2 to become active. As already described, the activation of TPCs is critical for maintaining proper lysosomal pH during starvation (Cang et al., 2013) and dissipating the luminal Na^+^ during the early hours of autophagy to allow amino acid buildup in the lysosomal lumen for subsequent mTORC1 reactivation, which represents an important step of autophagy termination. In this context, all channels and transporters involved in lysosomal pH regulation and/or amino acid efflux are also important for cellular responses under stressed conditions.

## PERSPECTIVES

In the traditional view, H^+^ is thought to be essential for lysosomal function because acidic pH is important for the degradation activities of lysosomal enzymes. As summarized in Table 1, however, the optimal pH values of lysosomal enzymes range from 3~7, and nearly half of them work optimally at pH values higher than 5. Therefore, the acidic pH, 4.5–5.0, of the lysosomal lumen normally reported for most mammalian cells may not be that important for many of the degradation enzymes. In fact, for most lysosomal enzymes, the pH-dependence curves are bell-shaped over a broad range and their activities do not alter dramatically with a small change in pH (Table 1). This questions the traditional view on degradation being the most critical function regulated by lysosomal pH. Given the critical role of luminal protons in the uptake of several other ions, including Ca^2+^, Cl^−^, and Na^+^, into the lysosome, it is worthwhile to consider that the acidic luminal pH also plays a pivotal role in maintaining the overall ion homeostasis of the acidic organelle.

Importantly, the luminal concentrations of Ca^2+^, Na^+^ and Cl^−^ are directly linked to several vital functions of the lysosome, including vesicle trafficking that requires both fission and fusion of the vesicles involved, enzymatic degradation and substance transport across the lysosomal membrane. As introduced earlier, the small volume of a lysosome makes it easy for luminal ionic composition to be perturbed by activities involving lysosomal ion channels and transporters. Thus, mechanisms have to be put in place to quickly bring the ionic concentrations back to normal for continued and/or next round of activities. The importance of H^+^ in maintaining luminal Ca^2+^ has been clearly established by numerous studies in which V-ATPase inhibitors were used to deplete lysosomal Ca^2+^ content (Christensen et al., 2002; Churchill et al., 2002; Calcraft et al., 2009). However, neither the mechanism by which Ca^2+^ leaks out of the lysosomes in response to luminal alkalinization nor the pathway for the H^+^-dependent Ca^2+^ uptake has been completely elucidated. The recent finding that P2X_4_ mediates lysosomal Ca^2+^ release under conditions when lysosomes are alkalinized (Cao et al., 2015a) provides a possible explanation of the former, but this may not be the only mechanism for the Ca^2+^ leak. A number of pathways actually favor Ca^2+^ release at acidic luminal pH, e.g. TRPML1 (Xu et al., 2007; Feng et al., 2014). In this regard, the dynamics of luminal pH may be important for differential regulation of activities of lysosomal Ca^2+^ release channels (Figure 3). It is also obvious that protons are needed for the function of NHE and ClC-6/7 to carry out the exchanger function to transport Na^+^ and Cl^−^. However, whether these are the only or even the main pathways for maintaining the homeostasis of these ions during ion channel activities warrants further investigation.

During activity, the change in ionic composition also affects lysosomal membrane potential, which significantly impacts ion fluxes across the lysosomal membranes. Thus, counter ions are always needed to help sustain the activity. It is intriguing that feed forward positive reinforcing loops exist to drive the system to continue its function. In the case of H^+^ uptake, Cl^−^ is believed to serve as the counter ion, where the acidified lysosome provides the H^+^ to drive the entry of Cl^−^ in a 1:2 ratio to neutralize the positive charge, which allows further uptake of H^+^ by the V-ATPase (Ishida et al., 2013). For TRPML1-mediated Ca^2+^ release, BK channel responds to the Ca^2+^ signal generated by TRPML1 and in turn mediates K^+^ entry to counter the lost positive charges due to Na^+^ and Ca^2+^ efflux. This activity allows further release of Ca^2+^ through the TRPML channel (Cao et al., 2015b). Therefore, lysosomal ion channels and transporter are intertwined to work in harmony to orchestra various lysosomal functions.

It is also important to emphasize that lysosomes are separate vesicles that function, typically, asynchronously in a given cell. Because of the spatiotemporal limits of the current microscopic techniques, the diversity in the dynamics and functional interactions of ion channels and transporters among individual lysosomes under given conditions are not really resolved under most experimental settings. The current research mostly relies on mobilizing the majority of the lysosomes towards one function through induction of receptor-mediated endocytosis, macropinocytosis, or autophagy so that synchrony is created somehow amongst most endolysosomes in the beginning hours (Yu et al., 2010; Grimm et al., 2014). Moreover, “road blocks”, such as inhibitors of V-ATPase, microtubule, and proteases, may be used to cause accumulation of intracellular vesicles at a certain stage of their function and alternations in such accumulation by pharmacological and/or genetics perturbation of a specific protein target will inform the functional significance of the protein. Using these approaches, strong evidence has emerged to suggest lysosomal ion channels and transporters as central regulators of lysosomal function.

The degradation function of lysosomes serves two main purposes: (i) to degrade internalized materials, including pathogens and signaling molecules such as epidermal growth factor, EGF (Sigismund et al., 2008); (ii) to degrade unwanted intracellular materials as a part of the autophagy pathway. To serve these purposes, efficient cargo delivery to lysosomes and prompt digestion are essential. Lysosomes contain more than 60 digestive enzymes, the activities of which are tightly dependent on lysosomal ion homeostasis maintained by ion channels and transporters. Indeed, defects in most lysosomal ion channels and transporters result in lysosomal storage just like that in glycosidases and sulfatases. In this context, the study showing impaired TRPML1 function in Niemann-Pick disease cells is particularly worth noting, as it revealed a new possibility that ion channel dysfunction underlies, at least partially, the Niemann-Pick pathology (Shen et al., 2012). Other studies also showed that upregulating TRPML1 channel function alleviated pathological phenotypes at the cellular level (Spampanato et al., 2013). Thus, ion channel dysfunction can be a major and common cause of pathogenesis in LSDs. As such, lysosomal ion channels may be potential therapeutic targets for many forms of LSDs. To fully realize this potential, further studies on lysosomal ion channels and their regulation by lipids and other cellular factors will be important. In conclusion, through regulating lysosomal ion homeostasis, lysosomal ion channels and transporters cooperate to provide an optimal environment for lysosomal digestive enzymes, regulate vesicle trafficking, and function as critical mediators of several essential signaling pathways including mTOR and TFEB to facilitate cell adaptation to metabolic stress.

## Figures and Tables

**Table 1:** Digestive enzymes in lysosomes

Name	pH optimum	Related disease	Reference
beta-Galactosidase-1/GLB1	3.5	GM1-gangliosidosis & Morquio B syndrome	(Zhang et al., 2000)
beta-Glucuronidase/GUSB	3.5	mucopolysaccharidosis type VII	(Shipley et al., 1993)
alpha-L-iduronidase/IDUA	3.5	mucopolysaccharidosis type I	(Scott et al., 1995)
Napsin A	3.5	–	(Aguilera et al., 2003)
Cathepsin D	3.5	–	(Barrett, 1977)
Cathepsin X/Z/P	3.5	–	(Nägler et al., 1999)
Chitobiase/CTBS	3.5	–	(Cacan et al., 1996)
alpha-Galactosidase A/GLA (rhGLA)	4	Fabry	(Ioannou et al., 1998)
a-N-acetylgalactosaminidase/NAGA	4	Schindler’s disease	(Wang et al., 1990)
Heparanase	4	–	(Vlodavsky et al., 1999)
Human hyaluronidase 1/HYAL1	4	mucopolysaccharidosis type IX	(Hofinger et al., 2008)
GM1-beta-galactosidase	4	gangliondosis	(Norden and O’Brien, 1975)
β-Galactocerebrosidase	4	Krabbe	(Martino et al., 2009)
Legumain/Asparaginyl endopeptidase	4	–	(Chen et al., 1997)
Galactosylceramidase/GALC	4.5	Krabbe’s Disease	(Suzuki and Suzuki, 1970)
alpha-N-acetylglucosaminidase/NAGLU	4.5	Sanfilippo syndrome B	(Schmidtchen et al., 1998)
alpha-L-fucosidase/FUCA1	4.5	fucosidosis	(Tiberio et al., 1995)
Hexosaminidase A/HEXA	4.5	Tay-Sachs disease	(Mahuran et al., 1988)
Human hyaluronidase 4/HYAL4	4.5	–	(Kaneiwa et al., 2012)
alpha-Glucosidase/GAA	4.5	Pompe’s disease	(Wan et al., 2008)
sialidase	4.5	Sialidosis	(Jung et al., 1989)
Cathepsin S	4.5	–	(Kirschke et al., 1989)
Arylsulfatase A/ARSA	4.5	metachromatic leukodystrophy (MLD)	(Lukatela et al., 1998)
Sphingomyelinase	5	Niemann Pick	(Mühle et al., 2013)
Iduronate 2-sulfatase	5	mucopolysaccharidosis II	(Wilson et al., 1990)
Glucosamine (N-acetyl)-6-sulfatase	5	mucopolysaccharidosis type IIID	(Robertson et al., 1992)
N-acetylgalactosamine-6-sulfatase	5	mucopolysaccharidosis type IVA	(Rivera-Colón et al., 2012)
Hexosaminidase B/HEXB	5.5	Sandhoff disease	(Korneluk et al., 1986)
Klotho	5.5	–	(Tohyama et al., 2004)
CathepsinA	5.5	–	(Jackman et al., 1990)
Cathepsin V	5.5	–	(Brömme et al., 1999)
Arylsulfatase G/ARSG	5.5	mucopolysaccharidosis (in mice)	(Ferrante et al., 2002)
Sulfamidase	5.5	mucopolysaccharidosis type IIIA	(Blanch et al., 1997)
Glucosylceramidase/GBA	6	Gaucher disease	(Sorge et al., 1985)
Acid ceramidase	6	Farber	(Bedia et al., 2010)
Chitotriosidase/CHIT1	6	–	(Aguilera et al., 2003)
Cathepsin B	6	–	(Krupa et al., 2002)
Cathepsin C	6	–	(McDonald et al., 1969)
Cathepsin L	6	–	(Mason et al., 1985)
Cathepsin O	6	–	(Mason et al., 1985)
Cathepsin H	6.5	–	(Barrett and Kirschke, 1980)
Cathepsin K	6.5	–	(Brömme et al., 1996)
Arylsulfatase B/ARSB	6.5	mucopolysaccharidosis Type VI	(Wicker et al., 1991)
AMSH/STAMBP	7.2	–	(McCullough et al., 2004)
beta-Glucosidase/GBA3	5~7	–	(de GRAAF et al., 2001)
Cathepsin E	3~7	–	(Athauda et al., 1991)
Cathepsin F	5.5~6.5	–	(Wang et al., 1998)
Lysosomal acid lipase	4	Wolman disease	(Dairaku et al., 2014)
